# Retraction Note: Functional modular networks identify the pivotal genes associated with morphine addiction and potential drug therapies

**DOI:** 10.1186/s12871-024-02482-0

**Published:** 2024-03-09

**Authors:** Yage Jiang, Donglei Wei, Yubo Xie

**Affiliations:** 1 Department of Anesthesiology, The First Affiliated Hospital of Guangxi Medical University, Nanning, 530021 China; 2 Department of Traumatology Orthopedic Hand Surgery, The First Affiliated Hospital of Guangxi Medical University, Nanning, 530021 China; 3 Guangxi Key Laboratory of Enhanced Recovery After Surgery for Gastrointestinal Cancer, The First Affiliated Hospital of Guangxi Medical University, Nanning, 530021 China

**Retraction Note: BMC Anesthesiology (2023) 23:151**

10.1186/s12871-023-02111-2

The Editor has retracted this article. After publication, concerns were raised regarding the datasets used in this study. Specifically, the dataset GSE30305 contains animals treated with heroin rather than with morphine. As such, the Editor no longer has confidence in the results and conclusions presented in the article.

None of the authors have responded to correspondence from the editor about this retraction.

